# Therapeutic Pearls at Random Strung

**Published:** 1881-06

**Authors:** 


### THERAPEUTIC PEARLS AT RANDOM STRUNG.

### ITCH.

Sponge the whole body, night and morning, with a solution of chloride of lime, or use an ointment composed of

℞ Liquid Storax, . . . . . ℥ iss.
Lard, . . . . . . . . ℥ ij.

Mix and strain. These applications will kill the acarus without irritating the skin.

### AGAIN,

℞ Olive Oil, . . . . . ʒ i.

Alcohol, . . . . . . ʒ ij.

Liquid Styrax, . . . . . ℥ i. Mix.

This mixture is to be thoroughly rubbed over the whole surface of the body, and to be repeated upon the following day. A general bath on the third day will remove the lotion and the dead acari.

### PERU BALSAM.

The value of this article in itch is of the highest character. I have personally tested it, after many other preparations had been used ineffectually.

Rub the Balsam into the skin, over, and considerably beyond, the eruption, so as to reach such animalculæ as may chance to be on the sound skin. Repeat the rubbing night and morning, and wash off in *five* days. In ten days the rubbing may be repeated if necessary. H. L. B.

ARISTOCRATIC REMEDY FOR ITCH.

| | | |
|---|---|---|
| ℞ | Balsam of Peru, . . . . | 1 ounce. |
| | Benozic acid, . . . . | 110 grains. |
| | Oil of cloves, . . . . | 40 drops. |
| | Alcohol, . . . . . | 2¼ drachms. |
| | Simple cerate, . . . . | 7 ounces. |

ITCH. (Army.)

Equal parts of glycerine and Olive Oil, mixed and rubbed in twice daily. Strict cleanliness, occasional bathing, and non-stimulating diet, constitute the best treatment for this very uncomfortable, and usually, intractable disease.

After washing the affected parts with a strong warm solution of common soda and allowing it to dry on the skin, apply freely paraffine. A few repetitions will accomplish all you need.

An almost specific for Army Itch may be found in a mixture of glycerine and corrosive sublimate, in the proportion of from three to five grains of the latter, to the ounce of the former, well rubbed in, night and morning. An occasional warm bath will facilitate the cure.

Take of simple cerate, ℥ i.; glycerine, ℥ ss.; subacetate of lead, ʒ i.; calomel ʒ i.; pulv. camphor, ʒ ss.; pulv. opium, ʒ i. Mix.

Rub on well three times per day for two or three days; then wash off well with castile or turpentine soap and water. One or two applications usually complete the cure.

Staphysagria, bruised and boiled in oil or lard, or mixed with glycerine and well rubbed in for three or four days, is a good remedy.

BARBER'S ITCH.

Stop shaving and keep the beard short with scissors; wash well night and morning with castile soap and soft water; after which rub the whole surface with the following ointment:

| | | |
|---|---|---|
| ℞ | Hydrarg. Chlor. Mit., . . . | ʒ i. |
| | Iodine (powdered), . . . . | ℈ i. |
| | Mix and add Adipis, . . . | ℥ i. |

Triturate until the composition assumes a red appearance. If a mild case use but little at a time, if a bad one, rub the sore thoroughly or until the whole is inflamed. Poultices of bread and milk, &c., may become necessary to remove scabs, &c. Iodide of Potash and sarsaparilla may become necessary when the disease becomes constitutional.

| | | |
|---|---|---|
| ℞ White Precipitate, | . . . . | ʒ i. |
| Ol. Trebinth, | . . . . . | ʒ i. |
| Simple cerate, | . . . . | ℥ i. Mix. |

The above recipe will be found most efficacious in Barber's Itch, old sores, and chronic cutaneous diseases generally.

